# “Opening Our Time Capsule”—Creating an Individualized Music and Other Memory Cues Database to Promote Communication Between Spouses and People With Dementia During Visits to a Nursing Home

**DOI:** 10.3389/fmed.2018.00215

**Published:** 2018-07-31

**Authors:** Ayelet Dassa

**Affiliations:** Music Department, Bar-Ilan University, Ramat Gan, Israel

**Keywords:** individualized music, music therapy and dementia, caregivers burden, nursing homes, spousal caregivers, family visits, communication in dementia

## Abstract

**Background:** Family members play a critical role in caring for people with dementia, and their involvement in care continues even after their loved ones are placed in long-term care facilities. The dynamics of family involvement following institutionalization are complex and challenging. The strain on caregivers does not cease and communication difficulties are a major barrier due to deteriorating language abilities as a result of dementia. Also, caregivers' involvement has implications on the quality of life of both the older adult and his family members.

**Objective:** To help alleviate caregivers' burden during visiting hours, by promoting communication opportunities. The program included the creation of an individualized database using personal music and photos that present life episodes.

**Methods:** A qualitative research was used to explore spouses' experience during visits and the process of creating and using the individualized database. Participants included three women who regularly visited their partners who had dementia and resided in a nursing home. The first phase included creating an individualized database for each couple. In the second phase, four meetings were conducted, each woman with her partner, utilizing the database on a tablet. A case study research design was used and various types of data were collected and analyzed. The data included interview reports (pre-post intervention), preparation meetings reports, spouses' recorded reactions at the end of each of the four visits, and the music therapist's written log during the program.

**Results:** All documented data revealed the difficulties, mostly the communication barrier, encountered by the three women during their visits to the nursing home. All reported that using the individualized database helped them to find ways to communicate with their partners, relive shared past experiences, and alleviate the stress and feelings of disconnection during visits.

**Conclusions:** Forming a bridge between past and present via individualized music and photos databases can be helpful in bridging the gap between people with dementia in nursing homes and their family members.

## Introduction

Dementia is undoubtedly one of the greatest global public health and social care challenges facing people today and, in the future, as stated in the 2015 World Alzheimer Report (1). The challenges of coping with dementia are multi-faceted and include family members as “partners in caregiving” (2). The burden of care mostly leads to nursing home placement, but the experience of family caregivers following nursing home placement receives less attention in research than the experience of caregivers providing home care (3). The myth that caregiving ends with placement in a nursing home is challenged by a critical review of studies, which shows that many families continue to remain involved in the lives of their loved ones, and the strain on caregivers does not cease after institutionalization (4–6). New challenges arise with placement in a nursing home, and family involvement programs should receive more attention (2, 4).

## Background and rationale

### Challenges of family visits in nursing homes

Although placement in a nursing home leads to reduced burden of care and some relief, it also evokes feelings of guilt and remorse among family members (7). The primary family member is the one most involved in the resident's life following institutionalization. Spouses who have longer relationship visit more often, and for longer duration, than adult children (6). Visiting can be stressful since communication difficulties are characteristic of people with dementia, and spouses who face this communication barrier show high rates of depression (8). Communication patterns between the caregiving wives and the care-receiving husbands was investigated by Braun et al. (9) among 37 couples. Findings revealed that caregiving wives used more neutral (e.g., problem discussion) and negative (e.g., hostility), and less positive (e.g., humor) communication than their husbands. Results also showed a strong positive relation between caring wives' mental health and their husbands' level of positive communication. Caregivers whose husbands used more positive communication reported less depression and distress (9).

### The use of music in promoting communication

A vast corpus of research deals with the impact of music on people with dementia (10–13). Music promotes engagement and social interaction and is particularly suitable for reminiscence (14, 15). Music helps to revive memories and rich association, tapping into long-term preserved memory (16). Despite memory loss, people with dementia can still take part in musical activities and enjoy singing old familiar tunes (17). The benefits of music are shown in various studies implementing music therapy and music-based interventions among caregivers and people with dementia: Music, and singing in particular, can help caregivers communicate with their care recipient, and alleviate agitation during daily care tasks (18). Reduced caregiver anxiety was found to be in parallel to decreased behavioral problems in a pilot project utilizing music therapy for people with dementia and their spouses. A joint music therapy group helped the caregiver to relax and enjoy (19). Self-reported relaxation was also shown in an exploratory study of a music program with family caregivers who enjoyed reminiscence and participating in musical activities with their loved ones with dementia at home (20). Also, everyday musical leisure activities, such as singing and listening to familiar songs, provided by the caregivers of people with dementia, was found beneficial to both parties and improved caregivers' well-being (21).

Music intervention programs for caregivers and people with dementia mostly relate to a home-based environment (20, 21). As previously stated, family members' engagement in care continues even after placement in a nursing home. The program's aim, presented in this manuscript, was to improve family members' experience during visits and help them to cope with the lack of communication by creating an individualized database comprised of specific “time capsule” episodes from the couple's life, based on their favorite music and other memory cues, such as photos and other related artifacts.

## Method

A qualitative research case study design was used to investigate a phenomenon within its real-life context (22), and embrace principles from participatory action research to empower the participants to take an active part in the intervention (23). The aim was to understand the experience of women who visited their spouses with dementia in a nursing home setting on a regular basis, following an intervention program using a personal database during visits. An in-depth description of such a social context might help to develop a practical intervention protocol suitable for family members who visit their loved ones in a nursing home setting. As Wheeler and Murphy (24) emphasized, several instrumental case studies can help to enhance the ability to theorize about a given phenomenon.

To understand the different aspects of the experience, two research questions were posed:

1. 1 What will be the influence of creating and using a personalized “time capsule” episodes on spouses' feelings toward visiting their partner in a nursing home?
2. 2 How will using the personalized database affect the relationship as perceived by the spouses?

### Target population

Three women who regularly visited their partners in a nursing home for people with dementia in Israel were recruited following an oral and written explanation given by the head social worker in the nursing home. All signed a consent form to include their material in this study. Names have been replaced by pseudonyms in this manuscript. All other data concerning personal information was concealed. The study was approved by the Bar-Ilan University Music Department's Ethics Committee (E.MUS.2017-2).

Personal information was gathered during the pre-intervention interview:

- Ruth (59), cared for her husband (60) who has had dementia for the past 4 years, and has resided in the nursing home for 6 months. Visit frequency: once a week for 2 hours.
- Sara (75), cared for her husband (78) who has had dementia for the past 10 years, and has resided in the nursing home for 1$\frac{1}{2}$ years. Visit frequency: once every 2–3 weeks, for half an hour.
- Miriam (76), cared for her husband (85) who has had dementia for the past 4 years, and has resided in the nursing home for 3 months. Visit frequency: three times a week, for 2 hours.

All three husbands were in middle stage dementia (As shown in a routine cognitive state assessment that took place in the nursing home every 3–6 months by the occupational therapist). They had no orientation in time or place, required full care in activities of daily living, and had speech difficulties.

### Overall framework

The research team included a social worker, a music therapist, and the researcher (a senior music therapist). All worked in the nursing home where the spouses visited.

The project was conducted in two phases:

The first phase included: 1. A personal interview with the social worker. The interview focused on the spouses' experience during visits and lasted approximately an hour; 2. Preparation of an individualized database with the music therapist. The database was comprised of music and other memory cues (photos, personal artifacts, etc.) that were uploaded to a tablet to provide easy access. The preparation lasted ~1 month and included phone calls, email correspondence, and meetings (varied individually); 3. An individual preparation meeting with the researcher, an hour long.

The second phase included: four meetings, each spouse with her partner, using the tablet during visits. The first two visits were accompanied by the music therapist, and the other two were conducted without guidance. The meetings took place according to individual visits' frequency, once a week or once in two-three weeks. Closing interviews were conducted approximately a week or two after completion, with the social worker, and a follow-up phone call was conducted by the researcher 2 months after the intervention.

### Procedure and data collection

#### Pre-intervention personal interview

The pre-intervention interview was conducted by the nursing home's social worker and included pre-defined questions regarding the frequency of visits, their feelings before, during, and after visits, their activities during visits, their feelings about communication options with their partner, whether they used music, photos, or any other games or instruments, whether they felt they lacked things to do, and believed it was possible to enhance communication with their partner during visits. The pre-intervention interview was summarized by the social worker, and was approximately an hour long.

#### Database creation process

At the end of the pre-intervention interview, the social worker instructed the spouses to start collecting relevant personal material, such as favorite music, personal photos from meaningful life events, and significant artifacts (special souvenirs, report cards, etc.). A few days following the interview, the music therapist met with each woman and further instructed her on how to collect material, focusing on 3–4 episodes from the past, that might elicit their partners' response. For example, if the spouse talked about the wedding and brought photos from that special event, the music therapist asked her about the music that was played during the wedding, and also whether she had the invitation or any other memorabilia, thus creating the “wedding story” that included photos, music, and artifacts.

The database creation process was individually tailored according to the women's needs and preferences. One woman needed more guidance and visited more often, so the music therapist met with her few times in the nursing home, while visiting her husband (3–4 half an hour meetings). In those meetings, the woman consulted the music therapist upon choosing adequate music and photos. Other visited less often, collected the material, and after one half an hour session with the music therapist, communicated further via e-mails and phone calls (no more than two short phone calls, and several e-mails, transferring music, photos, or names of musical pieces that the music therapist helped to search and added to the database). Overall, the preparation took a period of 1 month and included the music therapist's work uploading the data (music and photos) to an individualized file on the tablet.

Once the material was gathered and uploaded the researcher (the senior music therapist) met with each woman for an hour-long guidance session in which they decided together on what stories to focus and how to use the database. They were also instructed on how to communicate with their partner using the database: The researcher suggested various ways to encourage response, and useful ways of communication. They were advised to take a slow pace, not to rush, give their partner enough time to respond, avoid difficult questions that elicit anxiety, such as a “little quiz” testing their partners' memory. Instead, they were encouraged to tell the stories using the chosen music and photos as prompts. The preparation meetings were audiotaped for future analysis.

#### Visit meetings using the database

Each woman had four visits, matching her visits' frequency, once a week or once in two-three weeks, during which she used the personal database while visiting her husband. The women were asked to use the tablet at least 20 min during each visit. The music therapist joined the two first meetings and helped as necessary with incorporating the tablet. The other two meetings took place without the music therapist's guidance. After each of the four visits, the music therapist met with the women and recorded their brief spontaneous impression (3–5 min recording). In addition, the music therapist documented the process in a written report.

#### Post-intervention personal interview

The post-intervention interview included a conversation with the nursing home's social worker. It included some of the questions that appeared in the pre-intervention interview, such as frequency of visits, their feelings before, during, and after visits, and concluded questions about their experience during the program, whether they managed to communicate more, did they feel they received adequate training on using the tablet, whether something new happened, and whether they wanted to continue to use the tablet. The post-intervention interview was summarized by the social worker, and was approximately an hour long.

#### Follow up phone-calls

The researcher conducted a follow-up phone call approximately half an hour long, with each of the women, 2 months after the end of the process. The conversations were summarized by the researcher.

### Data analysis

A systematic qualitative content analysis (25) was conducted by the researcher, and it included all written reports documenting the process. Audio recordings were transcribed and analyzed. The material was analyzed step-by-step and it was formulated into content analytical units. The aspects of text interpretation following the research questions, were formed into categories, which were carefully formed and revised within the process of analysis. In order to avoid bias in the coding process, two colleagues (music therapists), who are not familiar with this work, observed the formed categories and shared their comments.

## Findings

Three women took part in this research and shared their experience with the researcher. Each woman took her own path during the project with regards to her emotional experience and her practical use of the tablet, yet similar characteristics were found. The content analysis of all the accumulated data revealed the similarities, and four categories emerged. The findings section will present a summary of each category:

### Visiting before the intervention was perceived as difficult and a communication barrier was evident

The spouses shared their difficulties and feelings regarding visiting times. Each coped with these feelings in her own way: “I have harsh feelings and thoughts about the place before I arrive. The most difficult thing for me is to overcome the obstacle and arrive […] it is very difficult for me to tolerate the physical state and the smell. I always ask the staff to prepare him before I arrive, and I prepare myself before entering” (Ruth, pre-interview); “I'm tense and it is not easy for me to be here. I disconnect myself emotionally. I have feelings of guilt that I'm free and he's not. I run quickly from here, escape from the smell in the ward. After visiting, I have feelings of worry and concern, whether he receives proper care, whether he's understood” (Sara, pre-interview); “If I'm not here with him, I'm o.k. I can have bad moments, but when I look at him, it's hard” (Miriam, pre-interview).

All indicated that they struggle to communicate with their partners, who have lost the ability to verbalize coherently. During visits, they try to communicate in various ways: “I bring him newspapers, coloring books, food that he likes, and I put on some of his favorite music on YouTube. He doesn't speak at all. No language” (Ruth, pre-interview); “I try to speak to him, he doesn't speak. I show him some photos of the children, he doesn't react” (Miriam, pre-interview); “I tell him about the children, about the weather. He seldom talks, has few words, not always clear. Sometimes he reacts, sometimes he doesn't, and then I go” (Sara, pre-interview).

### Creating the database evoked strong feelings and led to anticipation for the mutual meetings

All three women selected their partner's beloved musical tunes and meaningful photos from the family photo albums. The music therapist helped them to arrange the various materials and uploaded them into files on the tablet. Each file comprised a mini-story from the past, like the “trip to Europe”, or “dancing at weddings”. During this process, the women experienced strong emotions. Two of them talked about difficult times with their partners in the past and shared some hard feelings and reservations toward their partners. It also evoked feelings of pain, for example, one woman talked about her husband who no longer recognizes the son he was so proud of during their shared life. Nevertheless, the process and the meetings with the music therapist and the researcher also evoked positive emotions that empowered the women. Reminiscing while looking at the photos and listening to the music made them nostalgic. They found happiness looking at their kids' photos from years ago. They laughed and realized they also have some good memories. It helped them to feel more capable, as Sara concluded: “This is a great project! If I can get out of here without being so tense, it is already a big help” (Sara, preparation meeting). During the preparation meeting, the researcher advised against using difficult questions when communicating with their partner. Miriam laughed and admitted that she sometimes teases her husband to get a response: “I once told him I'm his sister and he got so annoyed with me. I just wanted to check that he knows who I am.” During the session, she realized she has so much to talk about with her husband. She was pleased and excited and looked forward to their meeting: “It never occurred to me I could tell him things from our past. Until now, I just asked him questions, and told him about things I'm currently doing. Now I have so many stories to tell him” (Miriam, preparation meeting).

Ruth, the youngest of them, reminisced during the preparation meeting: “we used to dance (looking at a beautiful photo of her with her husband at some wedding party) […] wow what a great tune (reacting to the music), this is him, this music is him!” (Ruth, preparation meeting). At the end of the meeting, she got so enthusiastic that she asked the music therapist to conduct the first mutual meeting right then, using the database: “This is going to replace the words that are gone. The strongest thing I feel right now is that I want to take him back home” (Ruth, preparation meeting).

### The mutual meetings using the database evoked positive feelings and hope

All three women had conducted four meetings, once a week, using the tablet. The procedure included the first two meetings with their husband and the music therapist, and the final two meetings were with their partners alone. They were asked to use the database for at least 15 min during their visit time. Miriam shared her experience: “I spoke slowly, and I didn't bombard him with questions, I talked about his family, and about things from our life. He was happy, and smiling, he didn't fall asleep. He was really looking at the photos. He listened and he understood, I know that. It is important that I sit there and talk to him. It was a really good experience” (Miriam, after the 3rd session). Sara was also excited and reacted positively after using the tablet: “I was very thrilled to see his excitement. I'm one hundred percent sure that his excitement was connected to what I showed him, it was a memory trigger […]. I saw that it really helped. For me, I felt much more relaxed and less tense. It also elicited some nice memories for me, thinking back about us with the children” (Sara, after the 4th session). The session format was flexible and allowed changes according to the participants' needs. Ruth, for example, expressed that she wanted the music therapist's support during the last session as well, after she tried one session (3rd session) alone with her husband. She had powerful meetings with her husband. For example, in the second meeting, Ruth mustered the courage and invited her husband to dance with her. She was so excited about the experience that she asked the music therapist to videotape them dancing together. Later on, she shared this moving clip with her friend and with her husband's brother who was considering not coming to visit since he felt that there was no longer any chance of communication. It was very powerful for her and she even called the researcher: “There was joy in here! Moments of happiness, like we used to have. I'm most grateful for that. It was only ten minutes, but I left here with a feeling I have never had before. Sparks of life that you can bring to people in here […]. I had an intimate experience with my husband. What happened here is amazing” (Ruth, after the 2nd session).

### Visiting after the intervention was perceived as feasible with the help of the database

The process of creating and using the database changed the way all three women perceived their visits. In the concluding interview, Ruth explained: “This project really helped change my feelings about visiting this place. It made me feel much happier with the meeting, it helped me to look back at the life we had, the feelings that were once a part of our emotional world. There was joy and movement, and a lot of smiles. It reflected the life we had before the illness.” She further added that the tablet helped her to communicate with her husband: “It illuminated memory fragments so we could do some fun things together. Music showed his strengths. Watching us dancing together made me understand why family members come every day. If only I could just spend time with him and the music; it is relaxing, fulfilling, calms things, and so much more” (Ruth, post interview).

Using the tablet made the spouses look forward to future visits. Sara considered buying her own tablet and adding more music, some current photos of the children, and things that would stimulate her partner's memory: “I think it really helped me. I had things to talk about. Usually when I talked with no specific context, I lost him, and he hardly listens. I learned that it is important to sit in a quiet place with him, and to adapt to a much slower pace. I'm much more relaxed, because I have something to do, I have a plan, and it alleviated the stress of my visits. I told the children they could use the tablet during their visits, and they need the training too” (Sara, post-interview). Miriam also felt much more competent and satisfied: “I learned how to talk to him. I can see he's really aware, alert and happy. It's very important I talk to him. I used to just come and say a few things. I knew from home that he liked to be talked to, but somehow, I stopped doing it. But now, thanks to the tablet, I see it does matter, that it's important for him. I want to add more photos and maybe some video clips of the children. Visiting with that tablet is much better than without it” (Miriam, post-interview).

## Discussion

The findings showed that spouses share difficulties in visiting their partner in a nursing home, as stated in several studies (4, 6, 8). Despite the fact that daily care is conducted by the nursing home staff, spouses still feel obligated to take care of their partner. As shown in a large sample study of long-term care families and residents, Cohen et al. (26) revealed that families of cognitively intact residents spend more time in activities related to social and community engagement, whereas families of residents with dementia spend more time on activities to support resident care.

The move to a nursing home creates a void in their role as a caregiver. They no longer have to deal with daily care tasks at home and assume a different role as visitors. This void might contribute to understanding the difficult feelings that accompany their weekly visits.

Participants in this project, despite having different spousal relationships, shared the burden of deterioration in dyadic communication due to the progressive state of the disease. Creating and using the individualized database helped them to form a bridge and overcome this barrier. Reviving their mutual past by opening “time capsule” episodes from their shared life, helped them to reconnect. Creating the database and participating in the project formulated aspects of their new role in their husbands' lives, and it might have helped in relinquishing feelings of guilt.

This new communication pathway helped them to understand that they are still needed for their partner's well-being. As one of the women kept saying: “it's very important I talk to him” (Miriam, post-interview).

It also helped to change their perception about visiting and caused them to experience their visits in the nursing home differently. They felt much more capable, it helped to create a framework for their visit, and define a personal space in an institutional environment, which robbed their privacy. One woman realized she could meet with her husband in a more private area when she visits, the other remembered their love of dancing and allowed herself to revive this mutual experience and dance with her husband. They no longer felt helpless during their visits, they could do something together and they could find ways to communicate.

Rewarding aspects of the caregiving relationship appear to be a major contributor to reducing depression among caregivers (27). Helping caregivers to cope and establish communication pathways between partners is a fundamental and critical task in the lives of people with dementia and their caregivers. Reciprocal interaction patterns positively affect a caregiver's well-being and mental health, and even affect quality of care. Research has shown that the degree to which a caregiver is depressed is directly related to how frequently they behaved in ways that might harm their care recipients (9). Therefore, developing therapeutic interventions to support communication during visits to a nursing home is crucial throughout the progression of the disease.

The database created a bridge between the couple, between past and present; a difficult present but a rich past with good memories, despite the complexity of the relationships at times. Music's ability to elicit both memories and emotions provided an important link to the individual's past and served as a means of nonverbal communication (10). Communicating through music helped both partners to engage in a different way that is not always accessible during family visits. Reliving mutual memories not only gave them a tool that facilitated communication, but it also helped them to revive their identity as a couple, after years of withdrawing into forced roles as caregiver and care recipient.

## Limitations and recommendations

The procedure presented in this case study research project required extensive preparation and included several meetings with the spouses, organizing the personalized database and conducting mutual meetings with both caregivers and care-recipients, in order to facilitate the use of the database via a tablet. The process of collecting these memories of music and photos following this procedure was very powerful for the spouses, but the main challenge of this project is to create a feasible procedure that will allow caregivers who come to visit the use of a personalized database. Although the individual path of each woman was important in collecting and developing the database, further research should develop according to the findings, a more structured and formalized procedure helping in the process of creating a personalized database. Developing assessment forms gathering data might be helpful in this process in order to facilitate more feasible procedure. Also, as shown there was a diversity between the women who took part in this research, concerning their need for closer involvement by the therapist. This raises important questions whether family members can use the tablet on their own or need constant support by the music therapist. Further development of this project should take into consideration this factor.

Another factor to consider is the potential obstacle of technology. Although using a tablet enabled easy and organized file preparation, it was not always comfortable for the spouses to use. Women who are not accustomed to technology should be provided with a hard copy database in a photo album format with adjacent CD.

Although promising, the findings should be interpreted with some caution, due to the small sample size and exploratory nature of this study. Further research is needed in order to understand the potential of using such a personalized database on a regular basis during visits, by spouses, family members or close friends. There were a few incidents throughout the project that involved other family members, like a surprise visit by one of the husband's sister, who joined a part of the meeting, and Ruth who came with her son for one meeting, and also shared the video clip of herself dancing with her husband, with family and friends. They all reacted positively to the idea of using the database, and further research is required.

## Author contributions

The author confirms being the sole contributor of this work and approved it for publication.

### Conflict of interest statement

The author declares that the research was conducted in the absence of any commercial or financial relationships that could be construed as a potential conflict of interest.
